# Knowledge user involvement is still uncommon in published rapid reviews—a meta-research cross-sectional study

**DOI:** 10.1017/rsm.2025.10018

**Published:** 2025-07-10

**Authors:** Barbara Nussbaumer-Streit, Dominic Ledinger, Christina Kien, Irma Klerings, Emma Persad, Andrea Chapman, Claus Nowak, Arianna Gadinger, Lisa Affengruber, Maureen Smith, Gerald Gartlehner, Ursula Griebler

**Affiliations:** 1 Department for Evidence-based Medicine and Evaluation, University for Continuing Education Krems, Cochrane Austria, Krems, Austria; 2 Cochrane Consumer Network Executive, London, UK; 3 RTI International, Research Triangle Park, NC, USA

**Keywords:** interst holder involvement, stakeholder involvement, evidence synthesis, rapid reviews

## Abstract

**Background:**

Involving knowledge users (KUs) such as patients, clinicians, or health policymakers is particularly relevant when conducting rapid reviews (RRs), as they should be tailored to decision-makers’ needs. However, little is known about how common KU involvement currently is in RRs.

**Objectives:**

We wanted to assess the proportion of KU involvement reported in recently published RRs (2021 onwards), which groups of KUs were involved in each phase of the RR process, to what extent, and which factors were associated with KU involvement in RRs.

**Methods:**

We conducted a meta-research cross-sectional study. A systematic literature search in Ovid MEDLINE and Epistemonikos in January 2024 identified 2,493 unique records. We dually screened the identified records (partly with assistance from an artificial intelligence (AI)-based application) until we reached the a priori calculated sample size of 104 RRs. We dually extracted data and analyzed it descriptively.

**Results:**

The proportion of RRs that reported KU involvement was 19% (95% confidence interval [CI]: 12%–28%). Most often, KUs were involved during the initial preparation of the RR, the systematic searches, and the interpretation and dissemination of results. Researchers/content experts and public/patient partners were the KU groups most often involved. KU involvement was more common in RRs focusing on patient involvement/shared decision-making, having a published protocol, and being commissioned.

**Conclusions:**

Reporting KU involvement in published RRs is uncommon and often vague. Future research should explore barriers and facilitators for KU involvement and its reporting in RRs. Guidance regarding reporting on KU involvement in RRs is needed.

## Highlights

### What is already known?

- Although knowledge user (KU) involvement can increase the relevance of rapid reviews (RRs) for decision-making, previous studies have shown that only 26%–43% of RRs involved KUs. Following the increased production of RRs in recent years, alongside new RR methods guidance emphasizing the importance of KU involvement, we wanted to know where we stand with KU involvement in RRs.

### What is new?

- KU involvement in RRs is still very uncommon and its reporting is limited. If an RR involved KUs, these were most often researchers/content experts or public/patient partners. They were most often involved at the beginning and the end of the RR process.

### Potential impact for RSM readers

- Our study highlights that KU involvement and its reporting in rapid evidence synthesis can still be greatly improved. Developing and evaluating methods for KU involvement that work within time and resource constraints would be essential to improve KU involvement in RRs. In addition, guidelines for RRs should emphasize the importance of reporting KU involvement.

## 1 Background

Rapid reviews (RRs) are a type of evidence synthesis that use modified systematic review (SR) methods to accelerate the review process and complete the review in a timely and resource-efficient manner.^1^ RRs are appealing to healthcare decision-makers because they can synthesize evidence quickly, particularly in urgent situations. Involving knowledge users (KUs) when conducting evidence syntheses is recommended to increase the review’s relevance.^2^^,^ ^3^ We define KUs as individuals who are likely to use the synthesized evidence to make informed decisions about clinical or health policy interventions or who might be affected by these decisions.^4^ By applying this definition, KUs can include individuals such as clinicians or other healthcare providers, health policymakers, patients, members of patient organizations, caregivers, or the general public.^2^

KU involvement is especially relevant in the context of RRs, as certain KUs often commission or request RRs. To make the RR as valuable and relevant as possible, close collaboration with the end user, e.g., the commissioner, is recommended by RR guidance.^5^^,^ ^6^ However, involving KUs is not yet the norm. Previous studies have shown KU involvement to range from 26% to 43% in RRs.^7^^-^ ^9^ In particular, the involvement of patients, who are primarily affected by health decisions but are usually not the commissioners of RRs, is often neglected. Notably, only 6% of the 103 RRs analyzed by Garritty et al. involved patients in the RR process.^8^

There are various methods and levels of engagement for involving KUs in the RR process. According to the Authors and Consumers Together Impacting on eVidencE (ACTIVE) framework,^10^ developed for SRs, KU involvement can be seen as a continuum from informing KUs about a review to involving them in one or more phases of the RR process (e.g., formulating the question and choosing the outcomes), or co-producing the complete review with them.^10^

While health policymakers and health organizations had already used RRs before the COVID-19 pandemic,^3^^,^ ^11^^-^ ^15^ the urgent need for evidence syntheses during the pandemic has worked as a catalyst for RRs, and the number of published RRs has increased tremendously. A PubMed search for articles with the term “rapid review” in the title retrieved approximately 250 records published until 2019, with more than 1,100 records published between 2020 and September 2024. In parallel to the increased demand for RRs, awareness of the importance of KU involvement has increased. While previous RR methods guidance has already recommended involving stakeholders in the RR process, such as commissioners, policymakers, and health system managers in the RR process,^5^^,^ ^6^ the RR methods guidance published online in October 2020 explicitly recommended involving a broad range of KUs, including patients and health professionals, throughout the whole RR process.^16^

### 1.1 Aim and research questions

We wanted to assess the proportion of KU involvement reported in recently published RRs (2021 onward). We also wanted to gain insight into which groups of KUs were involved in the specific phases of the RR process and the extent of their involvement. In addition, we wanted to explore whether the proportion of KU involvement was associated with certain factors. Specifically, we wanted to answer the following research questions:

1. What is the proportion of KU involvement reported in RRs published since 2021?
2. In what phases of RRs are KUs involved?
3. To what level are KUs involved in RRs?
4. Which KU groups are involved in RRs?
5. To what extent is KU involvement associated with other factors (*review type, year of publication, review topic, main rationale for choosing a rapid approach, RR protocol in place, citing an RR methods guidance, funding source, region of funding, and timeline of the RR*)?

## 2 Methods

### 2.1 Study design

We conducted a meta-research cross-sectional study to assess the proportion of KU involvement reported in RRs published since 2021. We chose this cut-off as new methods guidance for RRs was published online in October 2020, specifically recommending broad KU involvement,^16^ and due to the COVID-19 pandemic, the number of published RRs increased markedly. We published our protocol at the Open Science Framework (https://osf.io/gkm58/). When applicable, we adhered to the Strengthening the Reporting of Observational studies in Epidemiology (STROBE)^17^ reporting guideline throughout the manuscript.

### 2.2 Patient and public involvement statement

We involved a patient partner (MS) as a member of the project team, who provided input on the research questions, the data extraction form, the analyses and interpretation of data, and contributed to writing the manuscript.

### 2.3 Sample size calculation

We used a precision-based approach for sample size determination, where the aim was to enclose the exact two-sided 95% confidence limits for a binomial proportion (Clopper–Pearson method) within an interval at most 0.20 wide, regardless of the actual value of the proportion that we will observe in the sample.^18^^–^ ^20^ Therefore, we required a sample size of 104 RRs.

### 2.4 Identifying eligible RR

We conducted a systematic literature search in Ovid MEDLINE and Epistemonikos.org on January 09, 2024, to identify published RRs. We chose these two databases as we expected them to include most published RRs of studies in humans and health science. The search strategies are presented in detail in Appendix 1 of the Supplementary Material. Search terms were based on terminology identified by Hamel et al.^1^ To be eligible during the study selection phase, RRs had to use the term “rapid” in the title or abstract, had to be published in a peer-reviewed journal since 2021, and needed to be focused on a health-related topic. We were interested in any type of rapid evidence syntheses (e.g., RR of interventions, rapid qualitative evidence synthesis, rapid scoping review, etc.) published in any language and from any geographic region. We excluded publications that did not define themselves as rapid evidence syntheses, such as systematic reviews, and we excluded methods studies about RRs and protocols of RRs. We focused on RRs of studies in humans, as these are mostly used for decision-making in health care; therefore, we excluded RRs of laboratory or animal studies. When the RR was only part of a larger study and RR methods were not reported in detail, we also excluded the publication.

### 2.5 Study selection

We uploaded all identified records to DistillerSR.^21^ The use of this software was a deviation from the protocol, as we had originally planned to use Covidence and conduct all abstract/title screening dually and independently. Instead, we decided to use “DistillerSR Artificial Intelligence SYstem” (DAISY) integrated into DistillerSR to support study selection. We conducted a pilot exercise to calibrate the title/abstract screening among the whole team (B.N.S., U.G., C.K., D.L., L.A., E.P., A.G., and A.C.), using a sample of 50 records to screen.

At the title/abstract level, we screened 33% of the identified records dually by independent screeners. Because we had very high agreement in our inclusion/exclusion decisions (κ of 0.93), we switched to single human screening and AI confirmation. This means, the remaining 67% of records were screened by one person and the AI screening tool DAISY (part of the systematic review software DistillerSR). Disagreements between the human and the AI screener were resolved by the principal investigator (B.N.S.).

**Figure 1:**
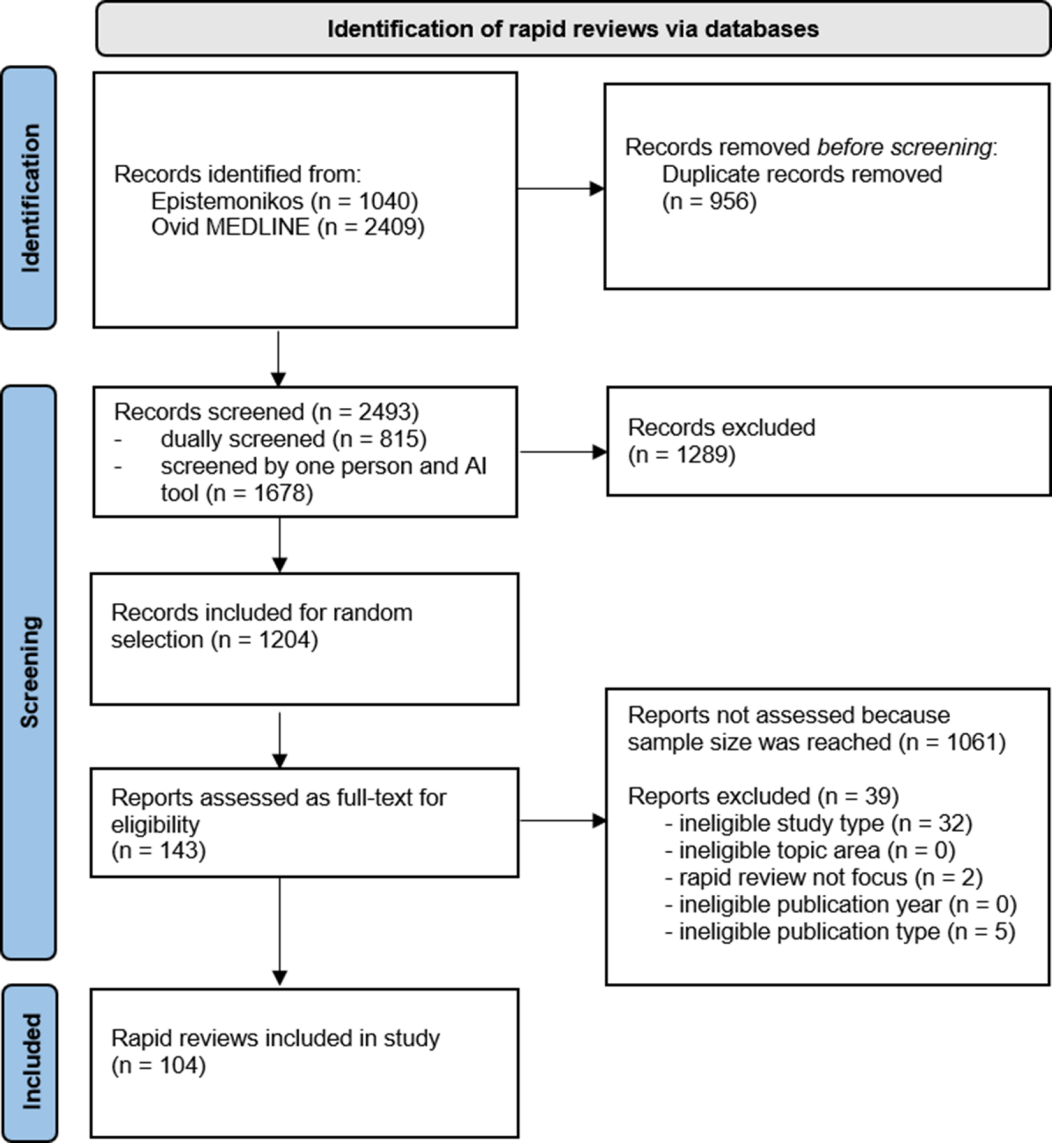
PRISMA 2020 Flow diagram.

We exported the list of all potentially relevant records to an Excel sheet and assigned computer-generated random numbers to each record. We then screened the full-texts dually and independently in order of their randomly assigned number until we reached 104 eligible RRs (see Figure 1).

### 2.6 Data extraction

We created a data extraction form in Excel. We pilot-tested the data extraction form on the same four RRs with all team members involved in extracting data. We discussed and clarified any uncertainties that arose during this phase and revised the form. After incorporating feedback from the piloting exercise, we conducted dual data extraction of the remaining 100 RRs (B.N.S., C.K., U.G., E.P., D.L., A.G., and A.C.). We extracted information on RR characteristics (first author’s name, year of publication, journal, type of RR, focus of the RR, rationale for choosing a rapid approach, RR protocol in place, citing an RR methods guidance, commissioner/funding, timeline of the RR) and if KU involvement was reported. If there were hints of KU involvement, but it was somewhat unclear (e.g., mentioning names in the acknowledgment section without specifying their role in the RR), we contacted the corresponding author of the RR and asked for clarification. When an RR reported KU involvement, we extracted to what extent different groups of KUs were involved for each phase of the RR and if any information on potential conflicts of interest of KUs was reported. Based on feedback from the patient partner (MS), we also extracted whether authors used a framework to report KU involvement, whether they indicated if KU involvement was mandatory (e.g., if a funder made it mandatory), whether KUs were compensated for their contributions, whether they were mentioned as co-authors, or whether the authors explicitly reflected on KU involvement.

### 2.7 Data analysis

Our primary outcome of interest was the proportion of KU involvement reported in RRs published since January 2021. We applied a broad definition of KU involvement. We considered KU involvement to be any involvement of at least one person who can be regarded as KU during at least one step of the review process. To define a KU, we considered any person who potentially uses synthesized evidence for decision-making in health care, comprising individuals such as clinicians, healthcare providers, health policymakers, commissioners, patients, members of patient organizations, caregivers, or the general public. As reporting of KU involvement was sometimes unclear, we focused on the proportion of *reported* KU involvement, which may differ from the actual KU involvement if not reported in the peer-reviewed paper. If an RR only reported names in the acknowledgment sections without clarifying their role in the RR process, we did not consider this as KU involvement. To calculate the proportion of KU involvement in RRs, we took the number of RRs with explicitly reported KU involvement of any kind and divided it by the total number of all 104 analyzed RRs.

We used a descriptive approach to summarize the KU groups that were involved and the intensity of KU involvement (e.g., one-time or regular involvement) in each RR phase (preparation [e.g., formulating the research question and deciding on outcomes], searching, study selection, data extraction, risk of bias assessment, synthesis, interpreting findings, communication of results).

To explore whether other factors were associated with KU involvement, we also chose a descriptive approach. We looked at the following factors: review type, year of publication, review topic, main rationale for choosing a rapid approach, RR protocol in place, citing an RR methods guidance, funding source, region of funding, and timeline of the RR. We calculated the proportion of RRs with KU involvement within these subgroups and compared it with the proportion of the general sample. We did not compute statistical tests as the nature of the analysis was exploratory and due to the small amount of data. All analyses were performed using Microsoft Excel.

Since the proportion of RRs with unclear KU involvement was considerable (*n* = 11), we contacted their authors and asked for clarification. Five of eleven authors replied to us. We used the provided information in a sensitivity analysis presented at the end of the results section. However, the main analyses for all research questions focused on reported KU involvement. We chose not to mix KU involvement reported in the publication and KU solely described via email, as we received responses from only five out of eleven contacted authors, and we did not contact the 70 authors of RRs without any mention of KU involvement.

## 3 Results

Out of 2,493 records identified by our literature search, we included 104 randomly selected RRs^22^^-^ ^125^ that fulfilled our eligibility criteria (see Figure 1; PRISMA 2020^126^).

### 3.1 Characteristics of included RRs

Our sample consisted of a diverse range of RR types, with the largest groups being RRs of interventions (31%; 32/104) and rapid scoping reviews (30%; 31/104). Most of the included RRs focused on research questions regarding noncommunicable diseases (33%; 35/104), COVID-19 (31%; 32/104), or healthcare delivery and systems (29%; 30/104). Over half of the RRs (53%; 55/104) did not report a rationale for conducting the evidence synthesis rapidly. Those giving a rationale mentioned “time constraints and the need to make an urgent decision” as the main reason for choosing a rapid approach (39%; 41/104). Half of the RRs had reported having a protocol in place, and 84% (87/104) cited RR methods guidance. Only 12% (13/104) of the RRs were commissioned; others reported general research funding (55%; 57/104). Commissioners or funding bodies were mainly from Europe (40%; 28/70), North America (29%; 20/70), and Australia/New Zealand (14%; 10/70). The median time from conducting the last search to submitting the RR for publication was 6 months, ranging from 0 to 31 months in 67 RRs reporting this information. The median time from the last search to the publication of the RR was 11.5 months, ranging from 1–49 months in 88 RRs reporting this data. For more details on the characteristics of the included RRs, see Table 1.

**Table 1:** Characteristics of included rapid reviews

Characteristics of RR	Number of RR (%)
*Review type (n = 104)*	
RR of intervention	32 (31)
RR of diagnostic test accuracy or prognosis	8 (8)
Rapid scoping review	31 (30)
Rapid qualitative evidence synthesis	13 (12)
Rapid realist review	3 (3)
Other rapid review (e.g., mixed-method, economic, overview)	17 (16)
*Year of publication (n = 104)*	
2021	51 (49)
2022	18 (17)
2023	30 (29)
2024	5 (5)
*Review topic (n = 104)*	
COVID–19	32 (31)
Other infectious diseases	3 (3)
Noncommunicable diseases	35 (33)
Healthcare delivery/system	30 (29)
Patient involvement/shared decision-making	4 (4)
*Main rationale for choosing a rapid approach (n = 104)*	
Time constraints/urgent decision-making need	41 (39)
Resource limitation (costs, personnel)	3 (3)
Fast-evolving research field	5 (5)
Not reported	55 (53)
*RR protocol in place (n = 104)*	
Yes (publicly available)	35 (34)
Yes (not publicly available)	18 (17)
No protocol	1 (1)
Unclear (no protocol mentioned)	50 (48)
*Citing an RR methods guidance (n = 104)*	
Yes	87 (84)
No	17 (16)
*Funding source (n = 104)*	
Explicitly commissioned	13 (12)
General research funding	57 (55)
No funding or not reported	34 (33)
*Region of funding/commissioning source (n = 70)*	
Africa	0 (0)
Asia	4 (6)
Australia/New Zealand	10 (14)
Europe	28 (40)
North America	20 (29)
South America	1 (1)
International	5 (7)
Not reported	2 (3)
*Timeline of RR*	Median (range) in months
Last search date to submission (*n* = 67)	6 (0–31)
Last search date to publication (*n* = 88)	11.5 (1–49)
Time for overall RR conduct explicitly reported (*n* = 8)	3.25 (1–6)

*Abbreviations*: COVID-19, coronavirus disease 2019; RR, rapid review; *n*, number of reviews.

### 3.2 Reported knowledge of user involvement in RRs

#### 3.2.1 Proportion of KU involvement

Of the 104 included RRs, 20 reported KU involvement. This results in a *proportion of reported KU involvement of 19%* (95% confidence interval [CI] 0.12–0.28). In additional 11% (11/104) of RRs, KU involvement was unclear. There were hints, such as names mentioned in the acknowledgments section, but no explicit description of KU involvement throughout the publication. In the remaining 70% (73/104) of RRs, KU involvement was clearly not mentioned. We contacted the authors of the 11 RRs with unclear KU involvement via email. Five clarified that they did involve KU, the other six did not respond.

For the following analysis, we focused on the 20 RRs that reported KU involvement in the publication.^25^^,^ ^30^^,^ ^31^^,^ ^40^^,^ ^41^^,^ ^48^^,^ ^49^^,^ ^57^^,^ ^59^^,^ ^75^^,^ ^83^^,^ ^85^^,^ ^89^^,^ ^97^^,^ ^99^^,^ ^103^^,^ ^109^^,^ ^115^^,^ ^119^^,^ ^120^ We considered the five additional RRs that reported KU involvement upon request^36^^,^ ^64^^,^ ^104^^,^ ^113^^,^ ^114^ in a sensitivity analysis at the end of the results section.

Table 2 gives more information on the characteristics of the RRs with reported KU involvement.

**Table 2:** Details of rapid reviews with reported knowledge user involvement

Author (year)	RR type	Focus of the RR	Rationale for rapid approach	Protocol	RR guidance cited	Funding region	Timeline (last search to publication of RR)	KU- framework; Mandatory KU involvement; CoI statements for KU	KUs compensated; KUs mentioned as authors; reflection on KU involvement	KU involvement during RR steps; KU groups involved
Barnett (2022)^119^	RR of intervention	Mental, behavioral or neurodevelopmental disorders	NR	Yes publicly available	Yes	United Kingdom	20 months	No; No; Yes	No; Yes; Yes	Multiple steps; Patient and/or public partners, healthcare professionals, policymakers, content experts/researchers
Bryant (2022)^59^	Rapid Scoping review	Mental, behavioral, or neurodevelopmental disorders	Time constraints/ decision-making need	Yes publicly available	Yes	Australia	28 months	No; No; No	No; No; No	Single step; Patient and/or public partners
Butow (2023)^103^	Rapid Scoping review	COVID–19	Time constraints/ decision-making need	NR	Yes	No funding reported	26 months	No; No; No	No; No; No	Single step; Healthcare professionals, patient, and/or public partners
Carroll (2021)^49^	Rapid Realist review	Patient involvement /shared decision-making	NR	NR	Yes	Canada	NR	No; No; No	No; No; Yes	Multiple steps; Content experts/researchers
Clyne (2022)^115^	Other types of RR	COVID–19	Time constraints/ decision-making need	Yes publicly available	Yes	Ireland	6 months	No; No; No	No; No; No	Single step; Policymakers, healthcare professionals
Corp (2023)^40^	Other types of RR	Mental, behavioral, or neurodevelopmental disorders	Time constraints/ decision-making need	Yes publicly available	Yes	United Kingdom	22 months	ACTIVE Framework; No, No	No; No; Yes	Multiple steps; Patient and/or public partners
Gentry (2021)^120^	Rapid Scoping review	Mental, behavioral, or neurodevelopmental disorders	Time constraints/ decision-making need	Yes (not publicly available)	Yes	United Kingdom	11 months	No; No; No	No; No; No	Multiple steps; Content experts/researchers
Ghidei (2022)^99^	RR of intervention	Healthcare delivery/system	Time constraints/ decision-making need	NR	No	Canada	16 months	No; No; No	No; No; No	Single step; Content experts/researchers
Gkiouleka (2022)^57^	Rapid QES	Healthcare delivery/system	NR	Yes (not publicly available)	Yes	United Kingdom	14 months	No; No; No	No; No; No	Single step; Content experts/researchers
Kadowaki (2021)^89^	RR of intervention	Other chronic diseases	NR	NR	Yes	Japan	9 months	No; No; No	Commissioner/funder was KU; Unclear; No	Multiple steps; Commissioner/funder
Karlsson (2023)^48^	Rapid QES	Patient involvement /shared decision-making	NR	Yes publicly available	Yes	Denmark	16 months	GRIPP II; No; No	No; Yes; Yes	Multiple steps; Patient and/or public partners
O’Reilly (2021)^97^	RR of intervention	COVID–19	Time constraints/ decision-making need	Yes publicly available	Yes	Ireland	3 months	No; Yes; No	No; No; No	Single step; Healthcare professionals
Palese (2023)^31^	Rapid QES	Healthcare delivery/system	NR	Yes (not publicly available)	Yes	No funding	15 months	No; No; No	No; Yes; No	Multiple steps; Content experts/researchers
Russell (2023)^30^	RR of intervention	Mental, behavioral, or neuro developmental disorders	Time constraints/ decision-making need	Yes publicly available	Yes	Australia	9 months	No; No; No	No; No; No	Multiple steps; Patient and/or public partners, content experts/researchers
Ryan (2023)^41^	Rapid QES	COVID–19	Time constraints/ decision-making need	Yes publicly available	Yes	International	26 months	No; No; No	No; No; No	Single step; Content experts/researchers
Stich (2023)^25^	RR of intervention	Mental, behavioral, or neuro developmental disorders	Time constraints/ decision-making need	NR	Yes	Canada	15 months	No; No; No	Funder/commissioner was KU; No; No	Multiple steps: Patient and/or public partners
Stirling (2021)^85^	Rapid Scoping review	Healthcare delivery/system	Fast-evolving research field	Yes publicly available	Yes	No funding reported	10 months	No; No; No	No; Yes; No	Multiple steps; Healthcare professionals
Toft (2022)^109^	Rapid Scoping review	Patient involvement /shared decision-making	NR	Yes (not publicly available)	Yes	Denmark	18 months	No; No; No	No; Unclear; No	All steps; Content experts/researchers
Upshaw (2021)^83^	RR of prognosis	COVID–19	NR	Yes publicly available	Yes	Canada	11 months	No; No; No	No; No; No	Single step; Content experts/researchers
Wingert (2021)^75^	RR of prognosis	COVID–19	Time constraints/ decision-making need	Yes publicly available	Yes	Canada	10 months	No; No; No	No; No; No	Multiple steps; Content experts/researchers

*Abbreviations*: ACTIVE, Authors and Consumers Together Impacting on eVidencE; CoI, conflict of interest; GRIPP, Guidance for Reporting Involvement of Patients and the Public; KU, knowledge user; NR, not reported; RR, rapid review; QES, qualitative evidence synthesis. Explanation: other type of review = e.g., economic review, mixed methods review

#### 3.2.2 Knowledge user involvement during RR phases

Seventy percent (14/20) of the RRs with KU involvement engaged KUs in the *preparation of the RR* to ensure the topic and research questions are relevant to the KU’s needs. Half of the RRs (10/20) involved KUs during *searching* to help with the development of the search strategy, find the best search terms, and ensure that all important sources were searched. Forty percent (8/20) of the RRs involved KUs during the *interpretation* of findings and *communication of results* (writing the report, disseminating the results). Only a small proportion (5%–15%) involved KUs during *study selection, data extraction, risk of bias assessment*, and *synthesis* (see Table 3).

**Table 3:** Involvement of knowledge users during the rapid review process

Rapid review	Knowledge user involvement during steps of the rapid review process							
			Study	Data	Risk of			
Author (year)	Preparation	Search	selection	extraction	bias assessment	Synthesis	Interpretation	Communication
Barnett (2022)^119^	Yes	Yes					Yes	Yes
Bryant (2022)^59^	Yes							
Butow (2023)^103^	Yes							
Carroll (2021)^49^		Yes					Yes	
Clyne (2022)^115^	Yes							
Corp (2023)^40^		Yes					Yes	
Gentry (2021)^120^	Yes							Yes
Ghidei (2022)^99^	Yes							
Gkiouleka (2022)^57^			Yes					
Kadowaki (2021)^89^	Yes			Yes		Yes		Yes
Karlsson (2023)^48^	Yes			Yes		Yes	Yes	Yes
O’Reilly (2021)^97^	Yes							
Palese (2023)^31^							Yes	Yes
Russell (2023)^30^	Yes	Yes					Yes	Yes
Ryan (2023)^41^		Yes						
Stich (2023)^25^	Yes	Yes						
Stirling (2021)^85^	Yes	Yes	Yes				Yes	
Toft (2022)^109^	Yes	Yes	Yes	Yes	Yes	Yes	Yes	Yes
Upshaw (2021)^83^		Yes						
Wingert (2021)^75^	Yes	Yes						Yes
Proportion of rapid reviews that involved knowledge users in this step	70% (14/20)	50% (10/20)	15% (3/20)	15% (3/20)	5% (1/20)	15% (3/20)	40% (8/20)	40% (8/20)

#### 3.2.3 Level of KU involvement

Reporting on the level of KU involvement was rather vague. Often, authors used general terms such as *working closely* or *being consulted* without further specifications of how frequently and in what way (e.g., via survey, workshops, meetings, written feedback) this was done. To determine whether involvement was continuous or only once during the project, we assessed whether KUs were involved in one, multiple, or all steps of the RR process. Forty percent (8/20) reported KU involvement *only during one step of the review process*, 55% (11/20) involved them in *multiple steps of the review process*, and 5% (1/20) involved the KUs *throughout the whole RR process* (see Table 3).

In 20% of RRs (4/20), the involved KUs were clearly part of the review author team. No publication reported financial compensation of the involved KUs; the involved KU was the commissioner in 10% of the RRs (2/20), so one can assume the KU could contribute during working hours. One RR provided a conflict-of-interest statement for KUs (see Table 2).

In 20% of RRs (4/20), authors explicitly reflected on KU involvement: One RR (5%) provided a lived experience commentary, one RR (5%) focused per se on the topic of patient involvement, and two (10%) described the value of involving KUs in the process. These two were the only ones that used a KU involvement tool (ACTIVE Framework^10^ or GRIPP II^127^) (see Table 2).

#### 3.2.4 KU groups involved

In 80% (16/20) of the RRs, only one KU group was involved. In the remaining 20% (4/20) RRs, multiple KU groups were involved.

Overall, 55% (11/20) involved *content experts/researchers* from across the relevant research fields; 35% (7/20) involved *patient and/or public partners* ranging from consumers and patients with lived experience to carers and local community organizations. In 25% (5/20) of the RRs, *healthcare professionals* were involved. Two RRs involved *policymakers* and one the *commissioner/funder*. When involving multiple KU groups, three RRs engaged “patient and/or public partners” along with “healthcare professionals,” “researchers/content experts,” or “healthcare professionals, policymakers, and researchers/content experts.” A fourth RR involved “healthcare professionals” along with “policymakers” (see Table 2).

#### 3.2.5 Factors associated with KU involvement in RRs

**Figure 2:**
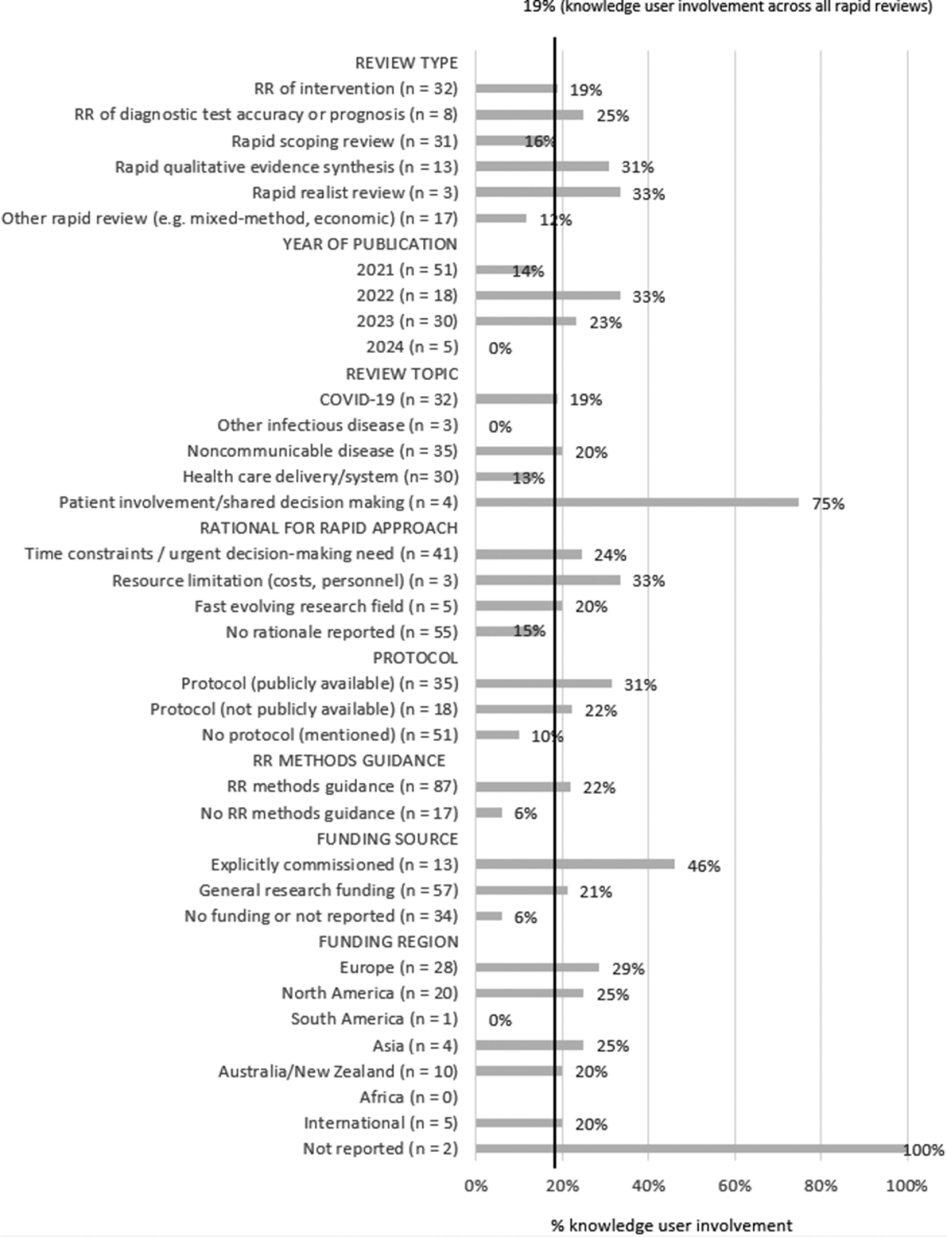
Reported knowledge user involvement per subgroup.

To determine if those RRs with KU involvement differed in any factors of interest, we calculated the proportion of KU involvement per subgroup (review type, year of publication, review topic, rationale for rapid approach, protocol, RR methods guidance, funding source, and funding country). As the general proportion of RRs with reported KU involvement was 19% in the sample, we considered this as the expected proportion (black line in Figure 2). As the number of RRs in the subgroups analyzed was often small, the results need to be interpreted with caution. Factors that might be associated with a higher proportion of KU involvement were review topics on patient involvement/shared decision-making and RRs that were explicitly commissioned. A lower proportion of KU involvement was seen in RRs that did not cite an RR methods guidance or reported no funding. For further details, see Figure 2.

When assessing the time from the last literature search to the submission of the RR, the median time for RRs with KU involvement was 7 months (range 1–26, *n* = 15), compared to 6 months (range 0–31, *n* = 67) in the general sample. When analyzing the time from the search to the final publication of the RR, the median time for RRs that involved KUs was 15 months (range 3–28; *n* = 19) compared to the general sample (median 11.5 months; range 1–49; *n* = 88).

#### 3.2.6 Sensitivity analysis

Authors of five RRs^36^^,^ ^64^^,^ ^104^^,^ ^113^^,^ ^114^ provided information on KU involvement upon request via email. We did not include this information in the main analysis as outlined in the methods section. To explore the impact of this decision on our research questions, we conducted sensitivity analyses adding these five RRs and calling it “clarified KU involvement.”

*Proportion of KU involvement.* When adding these five RRs, the proportion of “clarified KU involvement” was 24% (95% CI 0.16–0.33).

*Characteristics of RRs.* Of these five RRs, two were RRs of interventions, two were rapid scoping reviews, and one was categorized as “other RR type.” One focused on infectious disease, two on noncommunicable diseases, and two on healthcare delivery. Four of them cited RR methods guidance, three had a published protocol, and all five had funding. The time from the last search to publication ranged from 6 to 17 months.

*Phases and level of KU involvement.* When adding these five RRs, the proportion of KU involvement during the RR phases did not change markedly compared to the main analysis based on the 20 RRs. KU involvement was 68% during preparation (17/25), 56% during searching (14/25), 20% during study selection (5/25), 16% during data extraction (4/25), 8% during risk of bias assessment (2/25), 16% during synthesis (4/25), 48% during interpretation (12/25), and 48% (12/25) during communication.

Interestingly, four of these five RRs had KU listed as co-authors. One RR involved KU in all phases of the review, three in multiple phases, and one only in one phase.

*Groups of KU involved*. When adding these five RRs to the main analysis, the proportion of RRs involving content experts/researchers was still the largest at 48% (12/25), followed by patient and/or public partners at 40% (10/25), and healthcare professionals at 24% (6/25). None of these five RRs involved a funder or a policymaker.

**Figure 3:**
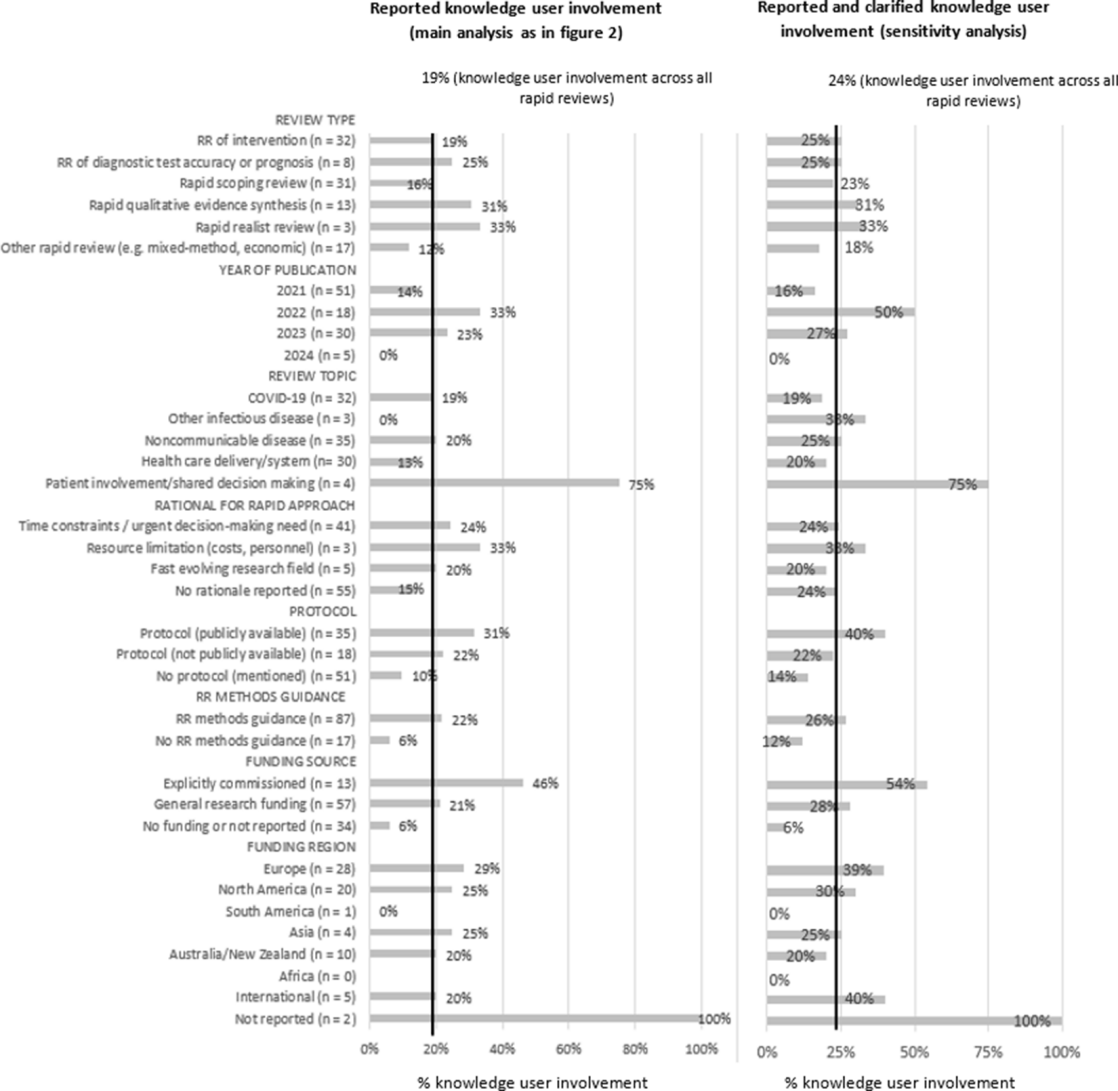
Knowledge user involvement per subgroup (main analysis vs. sensitivity analysis).

*Factors associated with KU involvement*. When adding these five RRs to the exploratory analysis of factors associated with KU involvement, we did not observe major changes to our main analysis (see Figure 3).

## 4 Discussion

Out of 104 RRs, only 19% (*n* = 20) reported KU involvement. This is lower than in previous studies, where KU involvement ranged from 26% to 43%.^7^^-^ ^9^ Additionally, 11% of the 104 assessed RRs had “unclear” KU involvement, with acknowledgments listing individuals but not clarifying their roles. When contacted, five authors replied, and all confirmed KU involvement that had not been reported in their RRs. This could be an indicator of potential underreporting of KU involvement and lack of awareness that KU involvement should be reported. In four of the five RRs, the KUs were part of the author team. Eventually, research teams considered the acknowledgment of RR contribution by co-authorship to be sufficient and that it did not need to be reported separately. In RRs with KU involvement, KUs typically contributed to early phases of the RR process (topic refinement, search), aligning with previous findings,^7^ or at the end, with interpreting and disseminating the findings. Although KU involvement is recommended throughout the whole RR process, it might be appropriate not to include some KU groups in all phases. For example, commissioners could have inherent interests or even conflicts of interest regarding specific findings and therefore should not be involved in the analysis part.

Garrity et al. found that while patient involvement was similarly reported in published and non-published RRs, commissioner involvement was underreported in published RRs.^8^ This may reflect why some RRs commissioned for urgent decision-making are not published in journals, or the strict word limit that most journals demand precludes authors from reporting KU involvement clearly. In our sample, one-third of the RRs were on COVID-19 and conducted for urgent decision-making needs, which may have precluded author teams from KU involvement. Time from the last search to submission of the publication was 1 month longer (median) in RRs with KU than in the general sample. While this can be perceived as a marginal difference in the overall review timeline, it can be significant in the context of urgent decision-making needs.

In our sample, content experts/other researchers were most often reported as KUs. It is possible that not all review teams were aware that involving other experts in the field could be considered as KU involvement. Patients and public partners were the second most often involved KU groups (*n* = 7 RRs). Similar results have been shown by Garritty et al.^8^ in a sample of published and non-published RRs from 2016. However, newer RR methods guidance^128^ explicitly recommends their involvement to ensure the RRs are relevant for those affected by health-care decisions.

Exploratory analysis indicated factors associated with higher KU involvement, namely a focus on patient involvement/shared decision-making, explicit commissioning, or having a published protocol. In contrast, RRs that did not cite any RR methods guidance or that reported no funding had less often reported KU involvement. These factors may serve as facilitators or barriers, though the small subgroup sizes caution against overinterpretation. Ongoing research, such as the Multi Stakeholder Engagement (MUSE) consortium’s work^129^ will shed further light on KU involvement by focusing exclusively on evidence syntheses with KU involvement, allowing for a larger sample size.

An interesting finding of our study was the diversity of RR types. Although originally focused on assessing intervention benefits and harms, many RRs are now being conducted as rapid scoping or qualitative evidence syntheses, reflecting a broader application of the RR process. This aligns with the evolving taxonomy of evidence synthesis, where “rapid” is considered a production mode rather than a specific review type.^130^

Our study, while comprehensive in its review of KU reporting across various RR types, has limitations. First, we focused on reported KU involvement, which may not capture actual involvement, as our findings suggest underreporting. This highlights the need for specific reporting guidelines for KU involvement in RRs. Second, we only analyzed published RRs, which may not fully reflect KU involvement in non-published RRs conducted for decision-making purposes. However, we wanted to assess the current situation in published RRs, as this is the product that is accessible to the research and decision-making community globally. Third, we used AI to assist with screening rather than dually screening all records. However, given the high agreement within the team and our random sampling approach, this likely had minimal impact on the results. Finally, we included only reviews explicitly labeled “rapid,” even though we searched using terms like “swift” or “targeted,” relying on the authors’ definitions of RR as we could not verify methods from abstracts alone. However, we assessed the methods of the included RRs and all applied at least one methods abbreviation compared to the traditional SR approach, so we are confident that our sample truly represented RRs.

Despite these limitations, our study provides valuable insights into the current state of KU involvement in RRs and highlights future research needs. Reporting remains scarce, likely due to the time and resource constraints inherent in RR processes and the strict word limits demanded by many journals. Many researchers may also not be aware that they should report KU involvement. Future research should explore barriers and facilitators to KU involvement in RRs, with a particular focus on the challenges posed by the rapid approach. A systematic review assessing challenges of public and patient involvement in evidence syntheses found influencing factors on both sides: the KUs and the researcher teams. One hindering factor on both sides was a lack of time.^131^ The MUSE consortium also plans to conduct a qualitative evidence synthesis on barriers and facilitators of KU involvement in systematic reviews.^129^ We would like to emphasize that there should be a specific attention to the assessment of KU involvement in the RR context, as factors like limited time and resources might differ in comparison to the traditional systematic review context. Developing and evaluating resource-efficient methods for KU involvement that work within time and resource constraints would also be essential to improve KU involvement in RRs. Future research should learn from best practice examples such as RR-producing entities like the COVID-END (COVID 19 Evidence Network to support Decision-Making) project^132^ and the SPOR (Strategy for Patient-Oriented Research) Evidence Alliance,^133^ which regularly include the public in the RR process. Additionally, to explore the actual extent of the underreporting of KU involvement or the perceptions and inherent definitions of authors, it would be interesting to assess KU involvement in a random sample via questioning the RR authors and comparing this information with the KU involvement reported in publications. Also, the forthcoming PRISMA-RR reporting guideline (OSF Link^134^) should include a specific item on KU involvement, detailing who was involved, at what phases of the review, and how. The broad range of RR types also indicates a need for future methods studies assessing the impact of RR methods on different types of rapid evidence synthesis, such as rapid scoping reviews or rapid qualitative evidence syntheses.

## Supporting information

Nussbaumer-Streit et al. supplementary material Nussbaumer-Streit et al. supplementary material

## Data Availability

The extracted data are available at https://osf.io/n2x9r.

## Supplementary material

To view supplementary material for this article, please visit http://doi.org/10.1017/rsm.2025.10018.

## References

[1] Hamel C , Michaud A , Thuku M , et al. Defining rapid reviews: a systematic scoping review and thematic analysis of definitions and defining characteristics of rapid reviews. J Clin Epidemiol. 2021;129:74–85. 33038541 10.1016/j.jclinepi.2020.09.041

[2] Garritty C , Tricco AC , Smith M , Pollock D , Kamel C , King VJ . Cochrane rapid reviews methods group. Rapid reviews methods series: involving patient and public partners, healthcare providers and policymakers as knowledge users. BMJ Evid Based Med. 2024;29(1):55–61. doi: 10.1136/bmjebm-2022-112070. PMC10850627 37076265

[3] Hartling L , Guise J-M , Hempel S , et al. Fit for purpose: perspectives on rapid reviews from end-user interviews. Syst Rev. 2017;6(1):32. 28212677 10.1186/s13643-017-0425-7 PMC5316162

[4] Canadian Institutes of Health Research. Knowledge user engagement. Accessed June 23, 2025. http://www.cihr-irsc.gc.ca/e/49505.html.

[5] Dobbins M. Rapid review guidebook: steps for conducting a rapid review. Accessed June 23, 2025. https://www.nccmt.ca/uploads/media/media/0001/02/800fe34eaedbad09edf80ad5081b9291acf1c0c2.pdf.

[6] Tricco A , Langlois E , Straus S , eds. Rapid Reviews to Strengthen Health Policy and Systems: A Practical Guide. World Health Organization; 2017.

[7] Feldmann J , Puhan MA , Mütsch M. Characteristics of stakeholder involvement in systematic and rapid reviews: a methodological review in the area of health services research. BMJ Open. 2019;9(8):e024587. 10.1136/bmjopen-2018-024587 PMC6701675 31420378

[8] Garritty C , Hamel C , Hersi M , et al. Assessing how information is packaged in rapid reviews for policy-makers and other stakeholders: a cross-sectional study. Health Res Policy Sys. 2020;18(1):112. 10.1186/s12961-020-00624-7 PMC7523380 32993657

[9] Griebler U , Dobrescu A , Ledinger D , et al. Evaluation of the interim Cochrane rapid review methods guidance—a mixed-methods study on the understanding of and adherence to the guidance. Res Synth Methods. 2023;14(6):824–846. 37483013 10.1002/jrsm.1656

[10] Pollock A , Campbell P , Struthers C , et al. Development of the ACTIVE framework to describe stakeholder involvement in systematic reviews. J Health Serv Res Policy. 2019;24(4):245–255. 30997859 10.1177/1355819619841647

[11] Mijumbi-Deve R , Rosenbaum SE , Oxman AD , Lavis JN , Sewankambo NK . Policymaker experiences with rapid response briefs to address health-system and technology questions in Uganda. Health Res Policy Sys. 2017;15(1):37. 10.1186/s12961-017-0200-1 PMC5415740 28468683

[12] Moore G , Redman S , Rudge S , Haynes A. Do policy-makers find commissioned rapid reviews useful? Health Res Policy Sys. 2018;16(1):17. 10.1186/s12961-018-0293-1 PMC5828139 29482643

[13] Patnode CD , Eder ML , Walsh ES , Viswanathan M , Lin JS . The use of rapid review methods for the U.S. preventive services task force. Am J Prev Med. 2018;54(1, Supplement 1):S19–S25. 29254522 10.1016/j.amepre.2017.07.024

[14] Peterson K , Floyd N , Ferguson L , Christensen V , Helfand M. User survey finds rapid evidence reviews increased uptake of evidence by Veterans Health Administration leadership to inform fast-paced health-system decision-making. Syst Rev. 2016;5(1):132. 27491354 10.1186/s13643-016-0306-5 PMC4974754

[15] Thigpen S , Puddy RW , Singer HH , Hall DM . Moving knowledge into action: developing the rapid synthesis and translation process within the interactive systems framework. Am J Community Psychol. 2012;50(3):285–294. 22777207 10.1007/s10464-012-9537-3 PMC4739647

[16] Garritty C , Gartlehner G , Nussbaumer-Streit B , et al. Cochrane rapid reviews methods group offers evidence-informed guidance to conduct rapid reviews. J Clin Epidemiol. 2021;130:13–22. 33068715 10.1016/j.jclinepi.2020.10.007 PMC7557165

[17] von Elm E , Altman DG , Egger M , Pocock SJ , Gøtzsche PC , Vandenbroucke JP . The strengthening the reporting of observational studies in epidemiology (STROBE) statement: guidelines for reporting observational studies. Int J Surg. 2014;12(12):1495–1499. 25046131 10.1016/j.ijsu.2014.07.013

[18] Brown LD , Cai TT , DasGupta A. Interval estimation for a binomial proportion. Stat Sci. 2001;16(2):101–133.

[19] Clopper CJ , Pearson ES . The use of confidence or fiducial limits illustrated in the case of the binomial. Biometrika. 1934;26(4):404–413.

[20] Newcombe RG . Two-sided confidence intervals for the single proportion: comparison of seven methods. Stat Med. 1998;17(8):857–872. 9595616 10.1002/(sici)1097-0258(19980430)17:8<857::aid-sim777>3.0.co;2-e

[21] DistillerSR Inc. DistillerSR. Accessed June 23, 2025. https://v2dis-prod.evidencepartners.com/Login/Login.php.

[22] Wang S , Wang S , Wang Y , Luan J . Glycemic control, weight management, cardiovascular safety, and cost-effectiveness of semaglutide for patients with type 2 diabetes mellitus: a rapid review and meta-analysis of real-world studies. Diabetes Ther. 2024;04:04. 10.1007/s13300-023-01520-3 PMC10838895 38175486

[23] Collins E , Philippe E , Gravel CA , Hawken S , Langlois MA , Little J. Serological markers and long COVID-A rapid systematic review. Eur J Clin Invest. 2024;54:e14149. 38083997 10.1111/eci.14149

[24] Favot K , Marnane V , Easwar V , Kung C. The use of telepractice to administer norm-referenced communication and cognition assessments in children with hearing loss: a rapid review. J Speech Lang Hear Res. 2024;67(1):244–253. 38016175 10.1044/2023_JSLHR-23-00354

[25] Stich C , Lakrouf R , Moreau J . Support interventions for young people in housing programs: a rapid literature review. J Prev. 2023;44(5):615–637. 10.1007/s10935-023-00743-1 37642906

[26] Youn BY , Cho H , Joo S , Kim HJ , Kim JY . Utilization of massage chairs for promoting overall health and wellness: a rapid scoping review. Explore (NY). 2023;05:05. 10.1016/j.explore.2023.10.002 37839928

[27] Krantz LM , Stanko-Lopp D , Ma MPH , Kuntz MJ , Wilcox HCP . A guide for schools on student-directed suicide prevention programs eligible for implementation under the STANDUP act, a rapid review and evidence synthesis. Arch Suicide Res. 2024;28(3):737–759. 37593936 10.1080/13811118.2023.2247033

[28] Ebrahim S , Blose N , Gloeck N , et al. Effectiveness of the BNT162b2 vaccine in preventing morbidity and mortality associated with COVID-19 in children aged 5 to 11 years: a systematic review and meta-analysis. PLOS Glob Public Health. 2023;3(12):e0002676. 38048340 10.1371/journal.pgph.0002676 PMC10695397

[29] Hendrikse C , Ngah V , Kallon, II , Leong TD , McCaul M . Ketamine as adjunctive or monotherapy for post-intubation sedation in patients with trauma on mechanical ventilation: a rapid review. Afr J Emerg Med. 2023;13(4):313–321. 38033380 10.1016/j.afjem.2023.10.002 PMC10682541

[30] Russell H , Aouad P , Le A , et al. Psychotherapies for eating disorders: findings from a rapid review. J Eat Disord. 2023;11(1):175. 37794513 10.1186/s40337-023-00886-w PMC10548609

[31] Palese A , Chiappinotto S , Bayram A , Sermeus W , Suhonen R , Papastavrou E. Exploring unfinished nursing care among nursing students: a discussion paper. BMC Nurs. 2023;22(1):272. 37596561 10.1186/s12912-023-01445-z PMC10436392

[32] Tomlinson OW . Predatory publishing in medical education: a rapid scoping review. BMC Med Educ. 2024;24(1):33. 38183007 10.1186/s12909-024-05024-x PMC10770935

[33] Roth L , Le Saux C , Gilles I , Peytremann-Bridevaux I. Factors associated with intent to leave the profession for the allied health workforce: a rapid review. Med Care Res Rev. 2024;81(1):3–18. 37864432 10.1177/10775587231204105 PMC10757398

[34] Dougherty L , Mathur S , Gul X , et al. Methods and measures to assess health care provider behavior and behavioral determinants in reproductive, maternal, newborn, and child health: a rapid review. Glob Health Sci Pract. 2023;11(Suppl 1):30. 10.9745/GHSP-D-22-00407 PMC10698233 38035722

[35] Broomfield M , Agabani Z , Guadagno E , Poenaru D , Baird R. The evidence mismatch in pediatric surgical practice. Pediatr Surg Int. 2023;39(1):295. 37978994 10.1007/s00383-023-05569-w

[36] Hibbert MP , Simmons R , Mandal S , Sabin CA , Desai M. A rapid review of antenatal hepatitis C virus testing in the United Kingdom. BMC Pregnancy Childbirth. 2023;23(1):823. 38017404 10.1186/s12884-023-06127-x PMC10683241

[37] Keegan D , Heffernan E , Clarke B , et al. Tools and methods for evaluating the change to health service delivery due to pandemics or other similar emergencies: a rapid evidence review. Eval Program Plann. 2024;102:102378. 37856938 10.1016/j.evalprogplan.2023.102378

[38] Heavner MS , Louzon PR , Gorman EF , Landolf KM , Ventura D , Devlin JW . A rapid systematic review of pharmacologic sleep promotion modalities in the intensive care unit. J Intensive Care Med. 2024;39(1):28–43. 37403460 10.1177/08850666231186747

[39] Brown TR , Xu KY , Glowinski AL . Structural racism and lessons not heard: a rapid review of the telepsychiatry literature during the COVID-19 public health emergency. Prim Care Companion CNS Disord. 2023;25(6):02. 10.4088/PCC.23r03563 PMC10666463 37923550

[40] Corp N , Bray L , Chew-Graham CA , et al. Self-directed self-management interventions to prevent or address distress in young people with long-term physical conditions: a rapid review. Health Expect. 2023;26(6):2164–2190. 37533152 10.1111/hex.13845 PMC10632640

[41] Ryan RE , Silke C , Parkhill A , et al. Communication to promote and support physical distancing for COVID-19 prevention and control. Cochrane Database Syst Rev. 2023;10:CD015144. 37811673 10.1002/14651858.CD015144 PMC10561351

[42] Paras ML , Searle EF , Lydston M , Shenoy ES . Alternate care site infection prevention and control practices: a rapid systematic review. Health Secur. 2023;21(4):286–302. 37311181 10.1089/hs.2022.0163

[43] Rodriguez Betancourt A , Samal A , Chan HL , Kripfgans OD . Overview of ultrasound in dentistry for advancing research methodology and patient care quality with emphasis on periodontal/peri-implant applications. Z Med Phys. 2023;33(3):336–386. 36922293 10.1016/j.zemedi.2023.01.005 PMC10517409

[44] Kapra O , Asna N , Amoyal M , Bashkin O , Dopelt K. The oncology clinical nurse specialist: a rapid review of implementation models and barriers around the world. Curr Oncol. 2023;30(8):7425–7438. 37623019 10.3390/curroncol30080538 PMC10453893

[45] Sanmarchi F , Fanconi C , Golinelli D , Gori D , Hernandez-Boussard T , Capodici A. Predict, diagnose, and treat chronic kidney disease with machine learning: a systematic literature review. J Nephrol. 2023;36(4):1101–1117. 36786976 10.1007/s40620-023-01573-4 PMC10227138

[46] Kamal A , Hodson A , Pearce JM . A rapid systematic review of factors influencing COVID-19 vaccination uptake in minority ethnic groups in the UK. Vaccines (Basel). 2021;9(10):01. 10.3390/vaccines9101121 PMC8541490 34696228

[47] McLure M , Macneil F , Wood FM , et al. A rapid review of burns first aid guidelines: is there consistency across international guidelines? Cureus. 2021;13(6):e15779. 34295589 10.7759/cureus.15779 PMC8291991

[48] Karlsson AW , Kragh-Sorensen A , Borgesen K , et al. Roles, outcomes, and enablers within research partnerships: a rapid review of the literature on patient and public involvement and engagement in health research. Res Involv Engagem. 2023;9(1):43. 37322525 10.1186/s40900-023-00448-z PMC10268359

[49] Carroll S , Kobayashi K , Cervantes MN , Freeman S , Saini M , Tracey S. Supporting healthy aging through the scale-up, spread, and sustainability of assistive technology implementation: a rapid realist review of participatory co-design for assistive technology with older adults. Gerontol Geriatr Med. 2021;7:23337214211023269. 34179298 10.1177/23337214211023269 PMC8202255

[50] Church G , Smith C , Ali A , Sage K. What is intensity and how can it benefit exercise intervention in people with stroke? A rapid review . *Front Rehabil Sci.* 2021;2:722668. 36188814 10.3389/fresc.2021.722668 PMC9397782

[51] Lieneck C , Bair J , Ardell S , Aldridge B , Austin BJ . Facilitators associated with nursing burnout in the ambulatory care setting as COVID-19 subsides: a rapid review. Healthcare (Basel). 2023;11(15):25. 10.3390/healthcare11152122 PMC10418695 37570363

[52] Thomas C , Ayres M , Pye K , Yassin D , Howell SJ , Alderson S. Process, structural, and outcome quality indicators to support perioperative opioid stewardship: a rapid review. Perioper Med (Lond). 2023;12(1):34. 37430326 10.1186/s13741-023-00312-4 PMC10332041

[53] Miskovic-Wheatley J , Bryant E , Ong SH , et al. Eating disorder outcomes: findings from a rapid review of over a decade of research. J Eat Disord. 2023;11(1):85. 37254202 10.1186/s40337-023-00801-3 PMC10228434

[54] Cipora E , Partyka O , Pajewska M , et al. Treatment costs and social burden of pancreatic cancer. Cancers (Basel). 2023;15(6):22. 10.3390/cancers15061911 PMC10047484 36980796

[55] Ramirez-Torres CA , Rivera-Sanz F , Sufrate-Sorzano T , Pedraz-Marcos A , Santolalla-Arnedo I. Closed endotracheal suction systems for COVID-19: rapid review. Interact J Med Res. 2023;12:e42549. 36548950 10.2196/42549 PMC9874988

[56] Sharma GD , Tiwari AK , Jain M , Yadav A , Srivastava M. COVID-19 and environmental concerns: a rapid review. Renew Sustain Energy Rev. 2021;148:111239. 34234623 10.1016/j.rser.2021.111239 PMC8189823

[57] Gkiouleka A , Aquino MRJ , Ojo-Aromokudu O , et al. Allied health professionals: a promising ally in the work against health inequalities- A rapid review. Public Health Pract (Oxf). 2022;3:100269. 36101762 10.1016/j.puhip.2022.100269 PMC9461647

[58] Indelicato AM , Mohamed ZH , Dewan MJ , Morley CP . Rapid antigen test sensitivity for asymptomatic COVID-19 screening. Primer. 2022;6:18. 35812789 10.22454/PRiMER.2022.276354 PMC9258726

[59] Bryant E , Spielman K , Le A , Marks P , Touyz S , Maguire S . Screening, assessment and diagnosis in the eating disorders: findings from a rapid review. J Eat Disord. 2022;10(1):78. 35672777 10.1186/s40337-022-00597-8 PMC9175461

[60] Chehade L , Zeitoun J , Lombe D , et al. COVID-19 vaccination in patients with cancer, a rapid review. Ecancermedicalscience. 2022;16:1355. 35510135 10.3332/ecancer.2022.1355 PMC9023301

[61] Schubbe D , Yen RW , Durand MA . How does patient socioeconomic position affect breast cancer surgical treatment and mortality?: a rapid review. Breast Cancer (Dove Med Press). 2021;13:595–601. 34737634 10.2147/BCTT.S293635 PMC8558100

[62] Hill JR , Brown JC , Campbell NL , Holden RJ . Usability-in-place-remote usability testing methods for homebound older adults: rapid literature review. JMIR Form Res. 2021;5(11):e26181. 34726604 10.2196/26181 PMC8596282

[63] Wingert A , Pillay J , Moore DL , et al. Burden of illness in infants and young children hospitalized for respiratory syncytial virus: a rapid review. Can Commun Dis Rep. 2021;47(9):381–396. 34650335 10.14745/ccdr.v47i09a05 PMC8448381

[64] Crowe S , Barker E , Roberts M , et al. Are we asking the right questions? Working with the LGBTQ+ community to prioritise healthcare research themes. Res Involv Engagem. 2021;7(1):64. 34556178 10.1186/s40900-021-00298-7 PMC8460395

[65] Lee N , Lee ES , Yun JM , et al. Behavioral therapy and pharmacotherapy for relapse prevention in abstinent smokers: a rapid review and meta-analysis for the Korea Preventive Service Task Force. Osong Public Health Res Perspect. 2021;12(4):244–253. 34465073 10.24171/j.phrp.2021.0017 PMC8408415

[66] Mojahed A , Brym S , Hense H , et al. Rapid review on the associations of social and geographical isolation and intimate partner violence: implications for the ongoing COVID-19 pandemic. Front Psychiatr. 2021;12:578150. 10.3389/fpsyt.2021.578150 PMC8076499 33927649

[67] Amani B , Amani B. Efficacy and safety of nirmatrelvir/ritonavir (Paxlovid) for COVID-19: a rapid review and meta-analysis. J Med Virol. 2023;95(2):e28441. 36576379 10.1002/jmv.28441 PMC9880713

[68] Cirillo N. Taste alteration in COVID-19: significant geographical differences exist in the prevalence of the symptom. J Infect Public Health. 2021;14(8):1099–1105. 34274859 10.1016/j.jiph.2021.07.002 PMC8266516

[69] Cardwell K , Jordan K , Byrne P , et al. The effectiveness of non-contact thermal screening as a means of identifying cases of Covid-19: a rapid review of the evidence. Rev Med Virol. 2021;31(4):e2192. 34260781 10.1002/rmv.2192

[70] Clement J , Maldonado AQ . Augmenting the transplant team with artificial intelligence: toward meaningful AI use in solid organ transplant. Front Immunol. 2021;12:694222. 34177958 10.3389/fimmu.2021.694222 PMC8226178

[71] Chiumento A , Baines P , Redhead C , Fovargue S , Draper H , Frith L. Which ethical values underpin England’s National Health Service reset of paediatric and maternity services following COVID-19: a rapid review. BMJ Open. 2021;11(6):e049214. 10.1136/bmjopen-2021-049214 PMC8189755 34103322

[72] Bellotti L , Zaniboni S , Balducci C , Grote G. Rapid review on COVID-19, work-related aspects, and age differences. Int J Environ Res Public Health. 2021;18(10):13. 10.3390/ijerph18105166 PMC8152775 34068101

[73] Arienti C , Lazzarini SG , Pollini E , Patrini M , Kiekens C , Negrini S. Effectiveness of rehabilitation interventions in adults with multi-organ dysfunction syndrome: a rapid review. J Rehabil Med. 2021;53(8):jrm00221. 34037239 10.2340/16501977-2846 PMC8638732

[74] Alem L , Bacque J , Guihenneuc J , Delelis-Fanien H , Mimoz O , Migeot V. Quality indicators development and prioritisation for emergency medical call centres: a stakeholder consensus. BMJ Open Qual. 2021;10(2):05. 10.1136/bmjoq-2020-001176 PMC8154933 34035128

[75] Wingert A , Pillay J , Gates M , et al. Risk factors for severity of COVID-19: a rapid review to inform vaccine prioritisation in Canada. BMJ Open. 2021;11(5):e044684. 10.1136/bmjopen-2020-044684 PMC8126435 33986052

[76] Sutton EL , Kearney RS . What works? Interventions to reduce readmission after hip fracture: a rapid review of systematic reviews. Injury. 2021;52(7):1851–1860. 33985752 10.1016/j.injury.2021.04.049

[77] Heath L , Carey M , Lowney AC , Harriss E , Miller M. Pharmacological strategies used to manage symptoms of patients dying of COVID-19: a rapid systematic review. Palliat Med. 2021;35(6):1099–1107. 33983081 10.1177/02692163211013255 PMC8189007

[78] McEvoy D , McAloon C , Collins A , et al. Relative infectiousness of asymptomatic SARS-CoV-2 infected persons compared with symptomatic individuals: a rapid scoping review. BMJ Open. 2021;11(5):e042354. 10.1136/bmjopen-2020-042354 PMC8098293 33947725

[79] Thompson JR , Risser LR , Dunfee MN , Schoenberg NE , Burke JG . Place, power, and premature mortality: a rapid scoping review on the health of women in Appalachia. Am J Health Promot. 2021;35(7):1015–1027. 33906415 10.1177/08901171211011388

[80] Furlong L , Serry T , Bridgman K , Erickson S. An evidence-based synthesis of instructional reading and spelling procedures using telepractice: a rapid review in the context of COVID-19. Int J Lang Commun Disord. 2021;56(3):456–472. 33844388 10.1111/1460-6984.12619 PMC8250683

[81] Balasubramanian M , Hasan A , Ganbavale S , Alolayah A , Gallagher J. Planning the future oral health workforce: a rapid review of supply, demand and need models, data sources and skill mix considerations. Int J Environ Res Public Health. 2021;18(6):12. 10.3390/ijerph18062891 PMC7999471 33808981

[82] Lange S , Medrzycka-Dabrowska W , Zorena K , et al. Nephrogenic systemic fibrosis as a complication after gadolinium-containing contrast agents: a rapid review. Int J Environ Res Public Health. 2021;18(6):15. 10.3390/ijerph18063000 PMC8001337 33804005

[83] Upshaw TL , Brown C , Smith R , Perri M , Ziegler C , Pinto AD . Social determinants of COVID-19 incidence and outcomes: a rapid review. PLoS ONE. 2021;16(3):e0248336. 33788848 10.1371/journal.pone.0248336 PMC8011781

[84] Chaabna K , Doraiswamy S , Mamtani R , Cheema S. Facemask use in community settings to prevent respiratory infection transmission: a rapid review and meta-analysis. Int J Infect Dis. 2021;104:198–206. 32987183 10.1016/j.ijid.2020.09.1434 PMC7518963

[85] Stirling E , Willcox J , Ong KL , Forsyth A. Social media analytics in nutrition research: a rapid review of current usage in investigation of dietary behaviours. Public Health Nutr. 2021;24(6):1193–1209. 33353573 10.1017/S1368980020005248 PMC10195628

[86] Cheruiyot I , Kipkorir V , Ngure B , Misiani M , Munguti J , Ogeng'o J. Arterial thrombosis in coronavirus disease 2019 patients: a rapid systematic review. Ann Vasc Surg. 2021;70:273–281. 32866574 10.1016/j.avsg.2020.08.087 PMC7453204

[87] Zolopa C , Hoj S , Bruneau J , et al. A rapid review of the impacts of “Big Events ” on risks, harms, and service delivery among people who use drugs: implications for responding to COVID-19. Int J Drug Policy. 2021;92:103127. 33549464 10.1016/j.drugpo.2021.103127 PMC7816610

[88] Jazayeri D , Heng H , Slade SC , et al. Benefits and risks of non-slip socks in hospitals: a rapid review. Int J Qual Health Care. 2021;33(2):09. 10.1093/intqhc/mzab057 33755121

[89] Kadowaki T , Yamamoto F , Taneda Y , et al. Effects of anti-diabetes medications on cardiovascular and kidney outcomes in Asian patients with type 2 diabetes: a rapid evidence assessment and narrative synthesis. Expert Opin Drug Saf. 2021;20(6):707–20. 33706621 10.1080/14740338.2021.1898585

[90] Haldar P , Reza-Paul S , Daniel RA , et al. A rapid review of pre-exposure prophylaxis for HIV in the Asia-Pacific region: recommendations for scale up and future directions. Sex Health. 2021;18(1):31–40. 33632382 10.1071/SH20058

[91] Jacups SP , Kinchin I. A rapid review of evidence to inform an ear, nose and throat service delivery model in remote Australia. Rural Remote Health. 2021;21(1):5611. 33601890 10.22605/RRH5611

[92] Estrada F , Atienzo EE , Cruz-Jimenez L , Campero L. A rapid review of interventions to prevent first pregnancy among adolescents and its applicability to Latin America. J Pediatr Adolesc Gynecol. 2021;34(4):491–503. 33561565 10.1016/j.jpag.2021.01.022

[93] Jennings W , Spurling G , Shannon B , Hayman N , Askew D. Rapid review of five years of Aboriginal and Torres Strait Islander health research in Australia - persisting under-representation of urban populations. Aust N Z J Public Health. 2021;45(1):53–58. 33522668 10.1111/1753-6405.13072

[94] Joseph-Williams N , Abhyankar P , Boland L , et al. What works in implementing patient decision aids in routine clinical settings? A rapid realist review and update from the international patient decision aid standards collaboration. Med Decis Making. 2021;41(7):907–937. 33319621 10.1177/0272989X20978208 PMC8474331

[95] Graber KM , Byrne EM , Goodacre EJ , et al. A rapid review of the impact of quarantine and restricted environments on children’s play and the role of play in children’s health. Child Care Health Dev. 2021;47(2):143–153. 33238034 10.1111/cch.12832 PMC7753247

[96] E OM , Byrne P , Walsh KA , et al. Immune response following infection with SARS-CoV-2 and other coronaviruses: a rapid review. Rev Med Virol. 2021;31(2):e2162. 32964627 10.1002/rmv.2162 PMC7536965

[97] O'Reilly A , Tibbs M , Booth A , Doyle E , McKeague B , Moore J. A rapid review investigating the potential impact of a pandemic on the mental health of young people aged 12-25 years. Ir J Psychol Med. 2021;38(3):192–207. 32912358 10.1017/ipm.2020.106 PMC7711353

[98] Posselt M , McIntyre H , Procter N. The impact of screen media portrayals of suicide on viewers: a rapid review of the evidence. Health Soc Care Community. 2021;29(1):28–41. 32716609 10.1111/hsc.13112

[99] Ghidei W , Montesanti S , Tomkow K , Silverstone PH , Wells L , Campbell S. Examining the effectiveness, acceptability, and feasibility of virtually delivered trauma-focused domestic violence and sexual violence interventions: a rapid evidence assessment. Trauma, Violence & Abuse. 2022;24(3):15248380211069059. 10.1177/15248380211069059 PMC10240651 35343335

[100] Schofield G , Dittborn M , Selman LE , Huxtable R. Defining ethical challenge(s) in healthcare research: a rapid review. BMC Med Ethics. 2021;22(1):135. 34587950 10.1186/s12910-021-00700-9 PMC8479723

[101] Ochola E , Andhavarapu M , Sun P , Mohiddin A , Ferdinand O , Temmerman M. The impact of COVID-19 mitigation measures on sexual and reproductive health in low- and middle-income countries: a rapid review. Sex Reprod Health Matters. 2023;31(1):2203001. 37294328 10.1080/26410397.2023.2203001 PMC10259330

[102] Khatib MN , Sinha A , Mishra G , et al. WASH to control COVID-19: a rapid review. Front Public Health. 2022;10:976423. 36033810 10.3389/fpubh.2022.976423 PMC9403322

[103] Butow P , Shaw J , Bartley N , et al. Vaccine hesitancy in cancer patients: a rapid review. Patient Educ Couns. 2023;111:107680. 36842287 10.1016/j.pec.2023.107680 PMC9951090

[104] Exley J , Gupta PA , Schellenberg J , et al. A rapid systematic review and evidence synthesis of effective coverage measures and cascades for childbirth, newborn and child health in low- and middle-income countries. J Glob Health. 2022;12:04001. 35136594 10.7189/jogh.12.04001 PMC8801924

[105] Boileau-Falardeau M , Contreras G , Gariépy G , Laprise C. Patterns and motivations of polysubstance use: a rapid review of the qualitative evidence. Health Promot Chronic Dis Prev Can. 2022;42(2):47–59. 35170930 10.24095/hpcdp.42.2.01 PMC8935897

[106] Dorward J , Gbinigie O , Cai T , et al. The protease inhibitor lopinavir, boosted with ritonavir, as treatment for COVID-19: a rapid review. Antiviral Therapy. 2021;25(7):365–376. 10.3851/IMP3385 33704086

[107] Niznik JD , Collins BJ , Armistead LT , et al. Pharmacist interventions to deprescribe opioids and benzodiazepines in older adults: a rapid review. Res Social Adm Pharm. 2022;18(6):2913–2921. 34281786 10.1016/j.sapharm.2021.07.012 PMC8836277

[108] Guerrero Aznar MD , Villanueva Guerrero MD , Cordero Ramos J , et al. Efficacy of diet on fatigue, quality of life and disability status in multiple sclerosis patients: rapid review and meta-analysis of randomized controlled trials. BMC Neurology. 2022;22(1):388. 36266639 10.1186/s12883-022-02913-w PMC9583472

[109] Toft BS , Rodkjaer L , Andersen AB , et al. Measures used to assess interventions for increasing patient involvement in Danish healthcare setting: a rapid review. BMJ Open. 2022;12(12):e064067. 10.1136/bmjopen-2022-064067 PMC9806071 36572495

[110] Klimas J , Hamilton MA , Gorfinkel L , Adam A , Cullen W , Wood E. Retention in opioid agonist treatment: a rapid review and meta-analysis comparing observational studies and randomized controlled trials. Syst Rev. 2021;10(1):216. 34362464 10.1186/s13643-021-01764-9 PMC8348786

[111] Ejegi-Memeh S , Sherborne V , Harrison M , et al. Patients’ and informal carers’ experience of living with mesothelioma: a systematic rapid review and synthesis of the literature. Eur J Oncol Nurs. 2022;58:102122. 35339776 10.1016/j.ejon.2022.102122

[112] Robinson M , Aventin Á , Hanratty J , et al. Nothing so practical as theory: a rapid review of the use of behaviour change theory in family planning interventions involving men and boys. Reprod Health. 2021;18(1):126. 34120630 10.1186/s12978-021-01173-0 PMC8201745

[113] Okoli GN , Rabbani R , Lam OLT , et al. Offloading devices for neuropathic foot ulcers in adult persons with type 1 or type 2 diabetes: a rapid review with meta-analysis and trial sequential analysis of randomized controlled trials. BMJ Open Diabet Res Care. 2022;10(3):e002822. 10.1136/bmjdrc-2022-002822 PMC9109018 35552238

[114] Geneen LJ , Kimber C , Doree C , Stanworth S , Shah A. Efficacy and safety of intravenous iron therapy for treating anaemia in critically ill adults: a rapid systematic review with meta-analysis. Transfus Med Rev. 2022;36(2):97–106. 35031197 10.1016/j.tmrv.2021.12.002

[115] Clyne B , Jordan K , Ahern S , et al. Transmission of SARS-CoV-2 by children: a rapid review, 30 December 2019 to 10 August 2020. Euro Surveill. 2022;27(5):2001651. 35115076 10.2807/1560-7917.ES.2022.27.5.2001651 PMC8815097

[116] Gardam O , Ferguson RJ , Ouimet AJ , Cobigo V. Measuring social isolation in older adults: a rapid review informing evidence-based research and practice. Clin Gerontol. 2023;46(4):478–497. 36755517 10.1080/07317115.2023.2170843

[117] Black GB , Boswell L , Harris J , Whitaker KL . What causes delays in diagnosing blood cancers? A rapid review of the evidence. Prim Health Care Res Dev. 2023;24:e26. 37039465 10.1017/S1463423623000129 PMC10156470

[118] Lobo EH , Abdelrazek M , Kensing F , et al. Technology-based support for stroke caregiving: a rapid review of evidence. J Nurs Manag. 2022;30(8):3700–3713. 34350650 10.1111/jonm.13439

[119] Barnett P , Steare T , Dedat Z , et al. Interventions to improve social circumstances of people with mental health conditions: a rapid evidence synthesis. BMC Psychiatry. 2022;22(1):302. 35484521 10.1186/s12888-022-03864-9 PMC9047264

[120] Gentry SV , Paterson BA . Does screening or routine enquiry for adverse childhood experiences (ACEs) meet criteria for a screening programme? A rapid evidence summary. J Public Health. 2022;44(4):810–822. 10.1093/pubmed/fdab238 34231848

[121] Watling D , Preece M , Hawgood J , Bloomfield S , Kolves K. Developing an intervention for suicide prevention: a rapid review of lived experience involvement Arch Suicide Res. 2022;26(2):465–480. 33073734 10.1080/13811118.2020.1833799

[122] Hamel C , Garritty C , Hersi M , et al. Models of provider care in long-term care: a rapid scoping review. PLoS ONE. 2021;16(7):e0254527. 34270578 10.1371/journal.pone.0254527 PMC8284811

[123] Piskulic D , McDermott S , Seal L , Vallaire S , Norris CM . Virtual visits in cardiovascular disease: a rapid review of the evidence. Eur J Cardiovasc Nurs. 2021;20(8):816–826. 34632501 10.1093/eurjcn/zvab084 PMC8524521

[124] Lunny C , Antony J , Rios P , et al. Safety and effectiveness of dose-sparing strategies for intramuscular seasonal influenza vaccine: a rapid scoping review. BMJ Open. 2021;11(9):e050596. 10.1136/bmjopen-2021-050596 PMC8450963 34535483

[125] Brooks SK , Greenberg N , Wessely S , Rubin GJ . Factors affecting healthcare workers’ compliance with social and behavioural infection control measures during emerging infectious disease outbreaks: rapid evidence review. BMJ Open. 2021;11(8):e049857. 10.1136/bmjopen-2021-049857 PMC8370838 34400459

[126] Page MJ , McKenzie JE , Bossuyt PM , et al. The PRISMA 2020 statement: an updated guideline for reporting systematic reviews. BMJ. 2021;372:n71. 33782057 10.1136/bmj.n71 PMC8005924

[127] Staniszewska S , Brett J , Simera I , et al. GRIPP2 reporting checklists: tools to improve reporting of patient and public involvement in research. Res Involv Engage. 2017;3(1):13. 10.1186/s40900-017-0062-2 PMC5611595 29062538

[128] Garritty C , Hamel C , Trivella M , et al. Updated recommendations for the Cochrane rapid review methods guidance for rapid reviews of effectiveness. BMJ. 2024;384:e076335. 38320771 10.1136/bmj-2023-076335

[129] Tugwell P , Welch V , Magwood O , et al. Protocol for the development of guidance for collaborator and partner engagement in health care evidence syntheses. Syst Rev. 2023;12(1):134. 37533051 10.1186/s13643-023-02279-1 PMC10394942

[130] JBI. JBI evidence synthesis taxonomy initiative. Accessed June 23, 2025. https://evidencesynthesistaxonomy.com/.

[131] Agyei-Manu E , Atkins N , Lee B , Rostron J , Dozier M , Smith M , McQuillan R. The benefits, challenges, and best practice for patient and public involvement in evidence synthesis: a systematic review and thematic synthesis. Health Expect. 2023;26(4):1436–1452. 37260191 10.1111/hex.13787 PMC10349234

[132] McMaster Health Forum. COVID-END - COVID-19 evidence network to support decision-making. Accessed June 23, 2025. https://www.mcmasterforum.org/networks/covid-end.

[133] SPOR Evidence Alliance. Summative evaluation: 2018–2023. Accessed June 23, 2025. https://sporevidencealliance.ca/.

[134] Stevens A , Tricco A , Straus S , et al. Developing PRISMA reporting guidelines for rapid reviews: a study protocol. Accessed June 23, 2025. https://osf.io/3jcpe.

