# Supplementary material for: Knowledge user involvement is still uncommon in published rapid reviews—a meta-research cross-sectional study
Source: Res Synth Methods. 2025 Jul 10;16(6):876–99. doi: 10.1017/rsm.2025.10018 (PMC12657652; doi:10.1017/rsm.2025.10018)
Supplement: Nussbaumer-Streit et al. supplementary material [file S1759287925100185sup001.docx]

# Appendix 1

## Search overview

| Database name and host (and coverage time) | Date searched | Hits |
| --- | --- | --- |
| Ovid MEDLINE(R) ALL 1946 to January 08, 2024 | 09.01.2024 | 2409 |
| Epistemonikos.org | 09.01.2024 | 1040 |
|  | **Total (before deduplication)** | **3449** |
|  | **Total (after deduplication)** | **2493** |

## Ovid MEDLINE

| **#** | **Searches** | **Results** |
| --- | --- | --- |
| 1 | (Rapid adj2 (review? or evidence)).ti,bt,kf. | 2359 |
| 2 | (Rapid adj6 (appraisal or assessment or synthes?s) adj6 (evidence or literature)).ti,bt,kf. | 150 |
| 3 | rapid review.ab. | 1570 |
| 4 | (Rapid adj (literature review or qualitative review or systematic review or evidence assessment or evidence review or evidence summary or evidence-based review or meta-review or response review or meta-analysis or metaanalysis)).ab. | 483 |
| 5 | ((abbreviated or accelerated) adj2 (review? or evidence)).ti,bt,kf. | 102 |
| 6 | ((abbreviated or accelerated) adj (review? or evidence)).ab. | 60 |
| 7 | ((abbreviated or accelerated) adj (literature review or qualitative review or systematic review or evidence assessment or evidence review or evidence summary or evidence-based review or meta-review or response review or meta-analysis or metaanalysis)).ab. | 4 |
| 8 | ((targeted or streamlined or pragmatic or mini) adj (literature review or qualitative review or systematic review or evidence assessment or evidence review or evidence summary or evidence-based review or meta-review or response review or meta-analysis or metaanalysis)).ti,bt,kf. | 170 |
| 9 | ((targeted or streamlined or pragmatic or mini) adj2 review).ti,bt,kf,ab. and (systematic or search*).mp. | 1207 |
| 10 | "Systematic Review"/ or "Review"/ | 3376751 |
| 11 | ((rapid or abbreviated or accelerated) adj2 (review or evidence or synthesis)).ti,bt,ab,kf. | 7738 |
| 12 | 10 and 11 | 2669 |
| 13 | or/1-9,12 | 5193 |
| 14 | limit 13 to yr="2021 -Current" | 2409 |

## Epistemonikos.org

| Search | Result |
| --- | --- |
| (title:("rapid review" OR "accelerated review" OR "abbreviated review" OR "targeted review" OR "mini review" OR "streamlined review" OR "pragmatic review" OR "rapid systematic review" OR "accelerated systematic review" OR "abbreviated systematic review" OR "targeted systematic review" OR "mini systematic review" OR "streamlined systematic review" OR "pragmatic systematic review" OR "rapid literature review" OR "accelerated literature review" OR "abbreviated literature review" OR "targeted literature review" OR "mini literature review" OR "streamlined literature review" OR "pragmatic literature review" OR "rapid evidence review" OR "accelerated evidence review" OR "abbreviated evidence review" OR "targeted evidence review" OR "mini evidence review" OR "streamlined evidence review" OR "pragmatic evidence review" OR "rapid evidence-based review" OR "accelerated evidence-based review" OR "abbreviated evidence-based review" OR "targeted evidence-based review" OR "mini evidence-based review" OR "streamlined evidence-based review" OR "pragmatic evidence-based review" OR "rapid evidence assessment" OR "accelerated evidence assessment" OR "abbreviated evidence assessment" OR "targeted evidence assessment" OR "mini evidence assessment" OR "streamlined evidence assessment" OR "pragmatic evidence assessment" OR "rapid meta-analysis" OR "accelerated meta-analysis" OR "abbreviated meta-analysis" OR "targeted meta-analysis" OR "mini meta-analysis" OR "streamlined meta-analysis" OR "pragmatic meta-analysis" OR "rapid metaanalysis" OR "accelerated metaanalysis" OR "abbreviated metaanalysis" OR "targeted metaanalysis" OR "mini metaanalysis" OR "streamlined metaanalysis" OR "pragmatic metaanalysis") OR abstract:("rapid review" OR "accelerated review" OR "abbreviated review" OR "targeted review" OR "mini review" OR "streamlined review" OR "pragmatic review" OR "rapid systematic review" OR "accelerated systematic review" OR "abbreviated systematic review" OR "targeted systematic review" OR "mini systematic review" OR "streamlined systematic review" OR "pragmatic systematic review" OR "rapid literature review" OR "accelerated literature review" OR "abbreviated literature review" OR "targeted literature review" OR "mini literature review" OR "streamlined literature review" OR "pragmatic literature review" OR "rapid evidence review" OR "accelerated evidence review" OR "abbreviated evidence review" OR "targeted evidence review" OR "mini evidence review" OR "streamlined evidence review" OR "pragmatic evidence review" OR "rapid evidence-based review" OR "accelerated evidence-based review" OR "abbreviated evidence-based review" OR "targeted evidence-based review" OR "mini evidence-based review" OR "streamlined evidence-based review" OR "pragmatic evidence-based review" OR "rapid evidence assessment" OR "accelerated evidence assessment" OR "abbreviated evidence assessment" OR "targeted evidence assessment" OR "mini evidence assessment" OR "streamlined evidence assessment" OR "pragmatic evidence assessment" OR "rapid meta-analysis" OR "accelerated meta-analysis" OR "abbreviated meta-analysis" OR "targeted meta-analysis" OR "mini meta-analysis" OR "streamlined meta-analysis" OR "pragmatic meta-analysis" OR "rapid metaanalysis" OR "accelerated metaanalysis" OR "abbreviated metaanalysis" OR "targeted metaanalysis" OR "mini metaanalysis" OR "streamlined metaanalysis" OR "pragmatic metaanalysis")) | 2210 |
| Filter: Systematic Review, Publication year:2021-2024 | 997 |
| Filter: Broad Synthesis, Publication year:2021-2024 | 43 |
| Total | 1040 |
| https://www.epistemonikos.org/advanced_search?q=(title:(%22rapid%20review%22%20OR%20%22accelerated%20review%22%20OR%20%22abbreviated%20review%22%20OR%20%22targeted%20review%22%20OR%20%22mini%20review%22%20OR%20%22streamlined%20review%22%20OR%20%22pragmatic%20review%22%20OR%20%22rapid%20systematic%20review%22%20OR%20%22accelerated%20systematic%20review%22%20OR%20%22abbreviated%20systematic%20review%22%20OR%20%22targeted%20systematic%20review%22%20OR%20%22mini%20systematic%20review%22%20OR%20%22streamlined%20systematic%20review%22%20OR%20%22pragmatic%20systematic%20review%22%20OR%20%22rapid%20literature%20review%22%20OR%20%22accelerated%20literature%20review%22%20OR%20%22abbreviated%20literature%20review%22%20OR%20%22targeted%20literature%20review%22%20OR%20%22mini%20literature%20review%22%20OR%20%22streamlined%20literature%20review%22%20OR%20%22pragmatic%20literature%20review%22%20OR%20%22rapid%20evidence%20review%22%20OR%20%22accelerated%20evidence%20review%22%20OR%20%22abbreviated%20evidence%20review%22%20OR%20%22targeted%20evidence%20review%22%20OR%20%22mini%20evidence%20review%22%20OR%20%22streamlined%20evidence%20review%22%20OR%20%22pragmatic%20evidence%20review%22%20OR%20%22rapid%20evidence-based%20review%22%20OR%20%22accelerated%20evidence-based%20review%22%20OR%20%22abbreviated%20evidence-based%20review%22%20OR%20%22targeted%20evidence-based%20review%22%20OR%20%22mini%20evidence-based%20review%22%20OR%20%22streamlined%20evidence-based%20review%22%20OR%20%22pragmatic%20evidence-based%20review%22%20OR%20%22rapid%20evidence%20assessment%22%20OR%20%22accelerated%20evidence%20assessment%22%20OR%20%22abbreviated%20evidence%20assessment%22%20OR%20%22targeted%20evidence%20assessment%22%20OR%20%22mini%20evidence%20assessment%22%20OR%20%22streamlined%20evidence%20assessment%22%20OR%20%22pragmatic%20evidence%20assessment%22%20OR%20%22rapid%20meta-analysis%22%20OR%20%22accelerated%20meta-analysis%22%20OR%20%22abbreviated%20meta-analysis%22%20OR%20%22targeted%20meta-analysis%22%20OR%20%22mini%20meta-analysis%22%20OR%20%22streamlined%20meta-analysis%22%20OR%20%22pragmatic%20meta-analysis%22%20OR%20%22rapid%20metaanalysis%22%20OR%20%22accelerated%20metaanalysis%22%20OR%20%22abbreviated%20metaanalysis%22%20OR%20%22targeted%20metaanalysis%22%20OR%20%22mini%20metaanalysis%22%20OR%20%22streamlined%20metaanalysis%22%20OR%20%22pragmatic%20metaanalysis%22)%20OR%20abstract:(%22rapid%20review%22%20OR%20%22accelerated%20review%22%20OR%20%22abbreviated%20review%22%20OR%20%22targeted%20review%22%20OR%20%22mini%20review%22%20OR%20%22streamlined%20review%22%20OR%20%22pragmatic%20review%22%20OR%20%22rapid%20systematic%20review%22%20OR%20%22accelerated%20systematic%20review%22%20OR%20%22abbreviated%20systematic%20review%22%20OR%20%22targeted%20systematic%20review%22%20OR%20%22mini%20systematic%20review%22%20OR%20%22streamlined%20systematic%20review%22%20OR%20%22pragmatic%20systematic%20review%22%20OR%20%22rapid%20literature%20review%22%20OR%20%22accelerated%20literature%20review%22%20OR%20%22abbreviated%20literature%20review%22%20OR%20%22targeted%20literature%20review%22%20OR%20%22mini%20literature%20review%22%20OR%20%22streamlined%20literature%20review%22%20OR%20%22pragmatic%20literature%20review%22%20OR%20%22rapid%20evidence%20review%22%20OR%20%22accelerated%20evidence%20review%22%20OR%20%22abbreviated%20evidence%20review%22%20OR%20%22targeted%20evidence%20review%22%20OR%20%22mini%20evidence%20review%22%20OR%20%22streamlined%20evidence%20review%22%20OR%20%22pragmatic%20evidence%20review%22%20OR%20%22rapid%20evidence-based%20review%22%20OR%20%22accelerated%20evidence-based%20review%22%20OR%20%22abbreviated%20evidence-based%20review%22%20OR%20%22targeted%20evidence-based%20review%22%20OR%20%22mini%20evidence-based%20review%22%20OR%20%22streamlined%20evidence-based%20review%22%20OR%20%22pragmatic%20evidence-based%20review%22%20OR%20%22rapid%20evidence%20assessment%22%20OR%20%22accelerated%20evidence%20assessment%22%20OR%20%22abbreviated%20evidence%20assessment%22%20OR%20%22targeted%20evidence%20assessment%22%20OR%20%22mini%20evidence%20assessment%22%20OR%20%22streamlined%20evidence%20assessment%22%20OR%20%22pragmatic%20evidence%20assessment%22%20OR%20%22rapid%20meta-analysis%22%20OR%20%22accelerated%20meta-analysis%22%20OR%20%22abbreviated%20meta-analysis%22%20OR%20%22targeted%20meta-analysis%22%20OR%20%22mini%20meta-analysis%22%20OR%20%22streamlined%20meta-analysis%22%20OR%20%22pragmatic%20meta-analysis%22%20OR%20%22rapid%20metaanalysis%22%20OR%20%22accelerated%20metaanalysis%22%20OR%20%22abbreviated%20metaanalysis%22%20OR%20%22targeted%20metaanalysis%22%20OR%20%22mini%20metaanalysis%22%20OR%20%22streamlined%20metaanalysis%22%20OR%20%22pragmatic%20metaanalysis%22))&protocol=no&min_year=2021&max_year=2024 | URL |
